# Anterior mediastinal neuroblastoma in an adult: an additional case of a rare tumor in an unusual location with review of the literature

**DOI:** 10.1186/s13000-023-01417-6

**Published:** 2023-11-29

**Authors:** Katrina Collins, Thomas M. Ulbright, Jessica L. Davis

**Affiliations:** Department of Pathology, Indiana University School of Medicine, 350 W 11th Street, Indianapolis, IN 46202 USA

**Keywords:** Adult, Anterior mediastinum, Neuroblastoma, Thymus

## Abstract

Neuroblastoma is rare in the adult population, especially in thoracic or mediastinal locations, with only 25 previously reported cases. We report an additional example of primary thymic neuroblastoma in a previously asymptomatic 71-year-old man with an anterior mediastinal mass who underwent robotic excision with pericardium and adjacent lung. The tumor was a 5.2 cm partially encapsulated, white-tan and rubbery mass with grossly identifiable areas of necrosis (25%) and hemorrhage. Histologically, the specimen showed a rim of adipose tissue and residual thymic tissue with areas of cystic thymic epithelium and prominent lymphoid tissue containing Hassall’s corpuscles. The tumor was composed of uniform, round cells with scant cytoplasm and small nuclei with inconspicuous nucleoli set within a background of conspicuous neuropil. Mitotic figures were easily found. By immunohistochemistry, the tumor cells expressed synaptophysin, chromogranin, NKX2.2 (diffuse, nuclear), GFAP (patchy), SMI31 (neurofilament) (focal, cytoplasmic), and TdT (diffuse, nuclear), while lacking expression of CD99, TTF-1, CK 20, MCPyV, PHOX2B, Olig2, OCT3/4, CD45, CD3 and PAX5. S100 protein was negative in the neuroblastic cells, with scattered positive cells in a vague sustentacular-like pattern. Fluorescence in situ hybridization for isochromosome 12p and *EWSR1* gene rearrangement were negative. As thymic neuroblastoma is extremely rare in adults, a neuroblastic tumor of germ cell origin (either primary or metastatic) or spread from a sinonasal tract tumor should be excluded because of differing treatments and prognoses. The properties of these rare neoplasms appear similar to olfactory neuroblastoma rather than pediatric-type neuroblastoma.

## Introduction

Neuroblastoma is the most common neurogenic tumor in children, typically occurring in infants and young children. The majority of cases are diagnosed prior to 5 years of age; rare cases are seen in older children and adults. Neuroblastomas arise from immature neural crest cells and have the potential to develop in various body sites, although they most commonly originate within the medulla of the adrenal gland. These tumors can also occur along the sympathetic chain/nerve ganglia, including the thoracic ganglia, a group of 12 paravertebral sympathetic ganglia in the posterior mediastinum, albeit less frequently as a primary site. Localized, posterior mediastinal neuroblastomas in young children have a more favorable prognosis in comparison to tumors occurring at other sites [1]. Adult-onset neuroblastomas are extremely rare, particularly in the anterior mediastinum/thymus, accounting for less than 1% of all neuroblastoma cases [2]. There are limited data detailing the biology and clinical course of these rare adult tumors, but they appear to exhibit distinct biological characteristics compared to pediatric or adolescent neuroblastomas and are associated with a less favorable prognosis [3]. Herein, we present a case highlighting the typical features of an anterior mediastinal neuroblastoma arising in an adult patient, along with a comprehensive review of existing literature since 1943.

## Case report

**Fig. 1:**
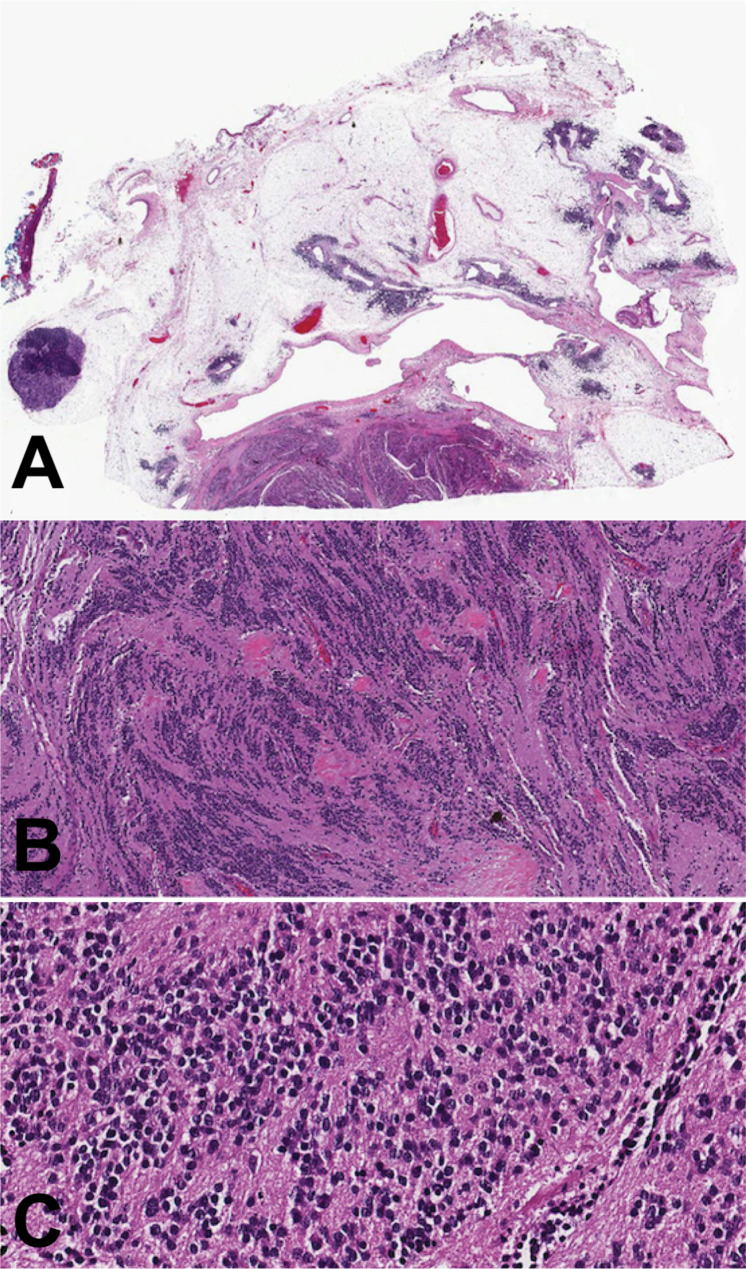
Microscopic images of the anterior mediastinal neuroblastoma. **A.** The tumor shows a rim of adipose tissue containing residual thymic tissue with areas of cystic thymic epithelium and prominent lymphoid tissue containing Hassall’s corpuscles. **B-C**. The tumor is composed of relatively uniform, round cells with scant cytoplasm and small nuclei with inconspicuous nucleoli set within a background of conspicuous neuropil

**Fig. 2:**
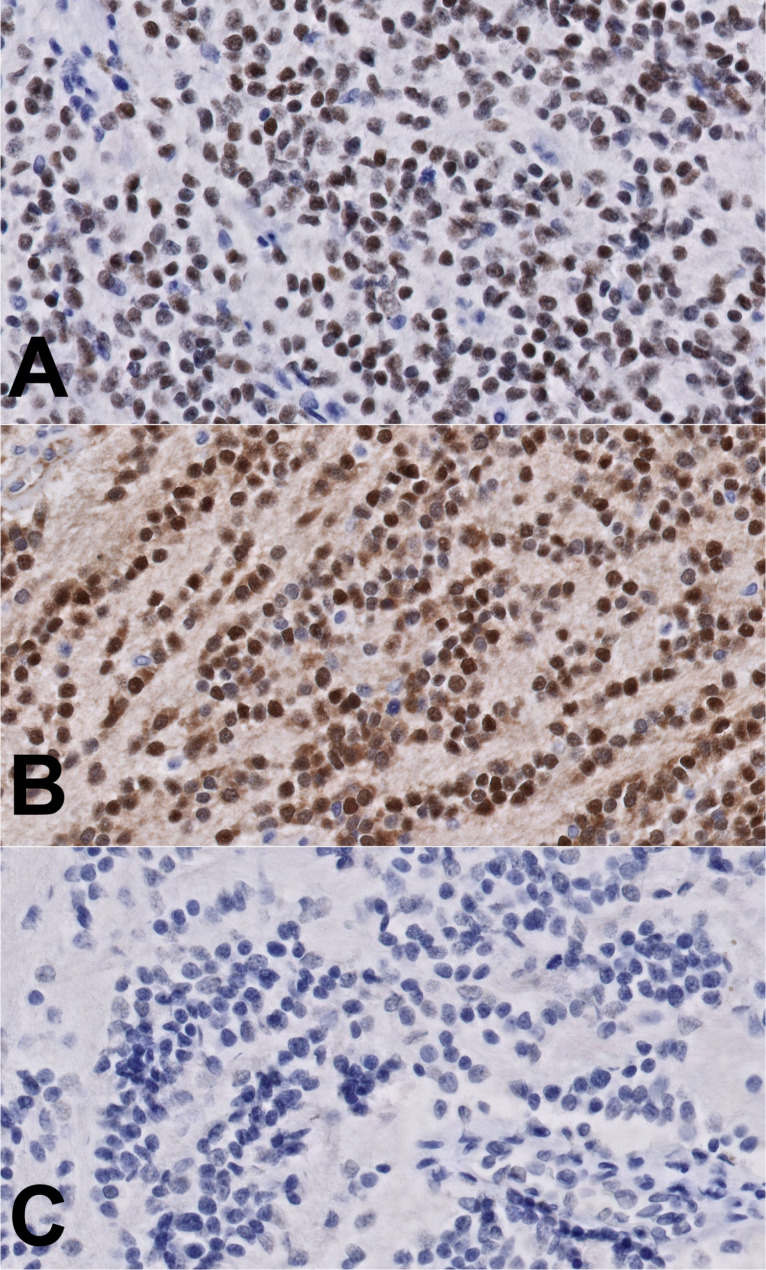
Immunohistochemical staining of the tumor cells. The tumor cells are positive for NKX2.2 (**A**) and TdT (**B**), while negative for PHOX2B (**C**)

The patient was an asymptomatic 71-year-old man with an anterior mediastinal mass who underwent robotic excision with pericardium and adjacent lung. The tumor was a 5.2 cm partially encapsulated, white-tan and rubbery mass with grossly identifiable areas of necrosis (25%) and hemorrhage. A minimal amount of loosely adherent adipose tissue was present. No evidence of syndrome of inappropriate antidiuretic hormone (SIADH) was reported, and the patient had no other known tumors. Histologically, the specimen showed a rim of adipose tissue containing residual thymic tissue (Fig. 1A) with areas of cystic thymic epithelium and prominent lymphoid tissue containing Hassall’s corpuscles. The tumor was composed of uniform, round cells with scant cytoplasm and small, round nuclei with inconspicuous nucleoli set within a background of conspicuous neuropil (Fig. 1B C). The tumor was identified near the ink of one soft tissue margin where there was disruption, which was considered to either represent a processing artifact or potentially a positive margin in that focal region. Mitotic figures were easily found (9–10 mitoses/10 high power fields). By immunohistochemistry, the tumor cells expressed synaptophysin, chromogranin, NKX2.2 (diffuse, nuclear) (Fig. 2A), GFAP (patchy), SMI31 (neurofilament) (focal, cytoplasmic), and TdT (Fig. 2B**)** (diffuse, nuclear), while lacking expression of CD99, TTF-1, CK20, MCPyV, PHOX2B (Fig. 2C**)**, Olig2, OCT3/4, CD45, CD3 and PAX5. S100 protein was negative in the neuroblastic cells, with scattered positive cells in a vague sustentacular-like pattern. Ki-67 proliferative index was 10–20%. Fluorescence in situ hybridization for isochromosome 12p and *EWSR1* gene rearrangement were negative. Post-operative serum tumor markers at 2 months were obtained and as follows: AFP 6.6 ng/mL, beta HCG less than 3 IU/L, and LDH 167 U/L. The patient continued surveillance imaging and showed no evidence of disease at 13 months of follow-up.

## Literature review

To our knowledge, to-date, there have been 25 cases of primary neuroblastoma in the anterior mediastinum, the majority of which were individual case reports, with a few case series (Table 1**)** [4–24]. Of the cases previously described, there was no gender predilection (males, 12 and females, 13). Our patient presented at a slightly older age than the mean age reported in the literature (71 years vs. 65 years, range 26 to 86 years; median 65 years). The tumor size in our case was slightly smaller than the mean size in prior reports (5.2 cm vs. 6 cm, range 3.4–12 cm; median 5.1 cm). Clinical presentations were as follows: sternal/chest pain (7, 1 also with severe myocardial infarction and 1 also with shortness of breath), asymptomatic (7), headache/dizziness/generalized fatigue/anorexia/nausea (6), hyponatremia (2), ataxia and oscillopsia (1), coronary artery disease (1), and not reported in 1 case. In 10 of 14 evaluated patients (71%) [8-11, 14, 16, 18, 20, 22, 23], serum levels of antidiuretic hormone were elevated (ranging from 2.1 to 6.4 pg/mL). Immunohistochemistry was performed as part of the initial diagnostic work up in 16 cases. In the majority of cases, immunostains for synaptophysin, chromogranin, CD56, and NSE showed positive expression, whereas epithelial markers were negative (i.e., CK AE1/AE3, Cam 5.2, EMA, CK 5/6), as well as CD99 and PHOX2B, when performed. One case, similar to our case, showed nuclear staining for TdT expression, although it was in a minor subset of tumor cells and not the diffuse positivity we found [23].

**Table 1:** Reports of anterior mediastinal neuroblastoma and ganglioneuroblastoma cases published between 1943– present (including our case)

Source	No. of cases	Age/Sex	Clinical presentation	Site	Size (cm)	Histology	Clinical evidence of SIADH, serum ADH level (pg/mL)	Immunohistochemistry		Molecular	Follow-up (mo/yr)
								Positive	Negative		
Sailer (1943)[4]	1	65/F	Headache, dizziness	AM	5	NB	NR	NP	NP	NP	Autopsy, incidental finding
Büthker et al. (1964) [5]	1	67/F	Sternal pain, incidental finding	AM	3.5	NB	NR	NP	NP	NP	ANED, > 12 mo
Hutchinson et al. (1968) [6]	1	51/M	Sternal pain, incidental finding	Thymus	10	NB	No	NP	NP	NP	ANED, 18 mo, adjuvant RT
Talerman et al. (1983) [7]	1	61/F	Chest pain, severe MI	Thymus	5.5	GNB	No	NP	NP	NP	Autopsy, incidental finding
Kaye et al. (1986) [8]	1	26/F	Nausea, anorexia, headache	AM, widespread deposits of metastatic tumor	NR	NB	Yes, 6.1	NP	NP	NP	DOD. 18 mo, adjuvant ChT/RT
Salter et al. (1995) [9]	1	80/F	Hyponatremia; history of renal cell carcinoma	AM	7	NB	Yes, NR	NSE, SYN, CG (few), S100 (sparse)	Vimentin, GFAP, CK AE1/AE3, Cam 5.2	NP	ANED, 14 mo
Asada et al. (1996) [10]	1	61/F	Fatigue and nausea	Thymus	4	GNB	Yes, 2.1	NSE, CG, S100 (ganglion cells), ADH (occasional), NF, SYN	VIP, NPY, SST	NP	NR
Argani et al. (1997) [11]	3	67/F	Coronary artery disease; history of endometrial and colonic carcinomas	Thymus	NR	GNB	Yes, NR	CG, SYN, NSE (Homer Wright rosettes and neuropil), Leu 7 (cases 1 and 3), neurofilament (case 3), S100 protein (spindled and dendritic cells, largely restricted to fibrovascular septa, prominent in cases 2 and 3)	CD99 (MIC2 gene product), GFAP, calcitonin, CK AE1/3, CAM 5.2	NP	DOC, POD 10
		80/M	NR	AM	7.3	NB	No			NP	ANED, 1.5 year after resection
		71/F	Asymptomatic	AM	NR	NB	NR			NP	DOD, 1 year after resection (local extension, distant metastases)
Nagashima et al. (1997) [12]	1	79/M	Chest pain	AM	8.0	GNB	NR	NSE and NF (small), S100 protein, HMB-45, (moderate-sized cells), NSE and S100 protein (pyramidal cells)			ANED, 5 year after resection
Tateishi et al. (2003) [13]	1	72/F	Asymptomatic	AM	NR	NB	NR	Vimentin (variable), CD56, CG, SYN, NSE, NF	CD99	NR	NR
Ogawa et al. (2009) [14]	1	60/M	Asymptomatic	Thymus, right lobe	4.7	NB	Yes, 6.4	NSE, SYN CG, ADH	NR	NP	NR
Ohtaki et al. (2011) [15]*	1	64/M	Asymptomatic	Superior mediastinum	5.0	NB	NR	NSE, SYN CG, CD56, vimentin	CK AE1/AE3, CK 5/6, EMA, E-cadherin, CD3, CD20, CD79a, CD117, S100 protein, SMA, CD99	*MYCN* amplified	AWD, lymph nodes metastases at 10 mo
Pellegrino et al. (2012) [16]	1	79/F	Progressive asthenia and severe hyponatremia	Thymus	12	NB	Yes, NR	NSE, SYN CG, CD56	NR	NP	ANED, 24 mo
Ueda et al. (2012) [17]	1	65/F	Asymptomatic	AM	6.4	NB	NR	SYN, CG, CD56	Keratin		ANED, 15 mo
Rogowitz et al. (2014) [18]	1	86/M	Fatigue, feeling “shaky”, shortness of breath	AM	5.2	NB	Yes, NR	SYN, CG, CD56, S100 (focal), NF (neuropil)	CK AE1/AE3, GFAP, CD99	FISH, *ESWR1* negative	ANED, 11 mo
Wiesel et al. (2015) [19]	1	62/M	Ataxia and oscillopsia	Thymus	7.5	NB	No	CG, SYN, NSE	CK AE1/AE3, CD99	*MYCN* non-amplified	ANED, 6 mo
Satoh et al. (2019) [20]	1	60/M	Asymptomatic	Thymus	4.7	NB	Yes, 6.4	NR	NR	NR	ANED, > 10 yr
Yanik et al. (2019) [21]	1	40/M	Anorexia, fatigue, headache, weight loss	AM (right paratracheal lesion); metastasis to hilar lymph nodes	NR	NB	NR	NR	NR	NR	ANED, 3 mo after resection with NACT
Watts et al. (2019) [22]	1	62/M	Non-specific constitution symptoms	Thymus	NR	NB	Yes, NR	CD56, SYN, CG, NSE, Ki67 (20%)	NR	NR	NR
Kennedy et al. (2022) [23]	1	83/M	Asymptomatic; history of prostatic adenocarcinoma	Thymus	3.6	NB	Yes, NR	CD56, CG, SYN (weak), NSE, ALK (weak), CD99, NeuN (variable), calretinin (variable), TdT and TTF1 (variable subset), SOX10 (rare), S100 (variable, rare), GFAP, PGP9.5	PHOX2B, panCK, Cam 5.2, EMA, CK 5/6, p40, NKX3.1, CD45, MyoD1, desmin, WT1, NF	FISH, *ESWR1* negative; NGS, diploid, 3q LOH, partial 3p loss (including *SETD2*)	
Moran et al. (2023) [24]	3	57–63/ F (2), M (1)	Chest pain, cough, shortness of breath	Thymus	3-4.5	NB	NR	SYN (neuropil), NSE	S100 protein, CG, SYN, CD99	NP	Alive (8–12 mo for 2 patients), Unknown for 1 patient
Current case	1	71/M	Asymptomatic	Thymus	5.2	NB	No	SYN, CG,TdT NKX2.2, GFAP (patchy), SMI31 (focal, cytoplasmic)	TTF1, CK 20, MCPyV, PHOX2B, Olig2, OCT3/4, CD99	FISH, *EWSR1* and i12p negative	Recent

* This case is described as occurring in the superior mediastinum, likely origin within overgrown thymus.

Abbreviations: AM, anterior mediastinum; ANED, alive with no evidence of disease; AWD, alive with disease; CAB, coronary artery bypass; ChT, chemotherapy; DOC, dead of other cause; DOD, dead of disease; FISH, fluorescence in situ hybridization; GNB, ganglioneuroblastoma; MI, myocardial infarction; NACT, neoadjuvant chemotherapy; NP, not performed; NR, not reported; POD, post-operative day; RT, radiation therapy; VF, ventricular fibrillation

Twenty-three patients had surgery initially. In addition, 3 of them received the following treatments: neoadjuvant chemotherapy (1 patient), adjuvant radiotherapy (1 patient); combination adjuvant chemotherapy and radiotherapy (1 patient). Two patients had tumor sampling at time of autopsy, without prior treatment. On follow-up of 17 patients, 11 were alive with no evidence of disease (follow-up range of 3 to 60 months; mean 16 months; median 13 months). One was alive with disease with lymph node metastasis at 10 months. Two were alive with unknown disease status at 8 and 12 months, and two died of disease at 12 and 18 months (1 with local extension and distant metastasis). One patient died of post-operative complications.

## Discussion

Primary mediastinal neurogenic tumors are a recognized occurrence, predominantly located in the posterior mediastinum in the pediatric population. They are believed to originate from the paravertebral sympathetic chain, which accounts for their frequent location in the posterior mediastinum. Moreover, during embryonic development, there exists the potential for sympathetic cells to migrate into the anterior mediastinum, with a particular tendency to establish within the thymus [25]. Although rare, similar tumors have been observed in adults, primarily limited to the anterior compartment [4–24], to which we now add this additional case.

Among mediastinal neurogenic tumors, neuroblastomas and ganglioneuroblastomas are prevalent in pediatric patients, whereas neurofibromas and ganglioneuromas are more frequent in adults. It is worth noting that rare ependymomas [26] and anterior mediastinal schwannomas have also been reported [27]. In one of the largest series [28], Adam and Hochholzer recorded 80 instances of ganglioneuroblastomas of the posterior mediastinum, utilizing data from the Armed Forces Institute of Pathology, with only three patients over 20 years old.

When considering mediastinal neuroblastomas in older patients, the outcomes are generally poor. Jrebi, et al. [29] reported 15 patients with ages ranging from 19 to 33 years. Of these, two had neuroblastomas located in the mediastinum (exact compartment not specified). In one patient, the tumor involved both the retroperitoneum and mediastinum. Five of the 15 patients were asymptomatic, and their tumors were incidentally discovered during imaging studies. Roughly half had advanced stage disease, characterized by metastatic tumors and poor prognoses. In contrast, those patients in early stages of the disease had better outcomes, with survival periods extending up to 9 years. Kaye, et al. [8] conducted a smaller series involving adult patients with mediastinal neuroblastoma, encompassing four individuals, two of whom had anterior mediastinal tumors. While there are limited cases with extensive follow-up, cases of neuroblastoma primary to the anterior mediastinum appear to have a superior outcome than neuroblastomas arising in other locations in adults (Table 1).

Clinically, patients who have been documented with anterior mediastinal or thymic neuroblastomas have typically manifested non-specific symptoms and were over 50 years old. In documented cases, 10 of 14 patients (71%) [8–11, 14, 16, 18, 20, 22, 23] manifested abnormal secretion of antidiuretic hormone. Interestingly, olfactory neuroblastomas, similar to mediastinal and thymic neuroblastomas, can be linked to the SIADH [30–35], unlike classic pediatric-type neuroblastoma, which is not associated with the syndrome.

It is essential to underscore establishing a diagnosis of anterior mediastinal or thymic neuroblastoma can pose a challenge. When an adult patient presents with an anterior mediastinal mass, it strongly points to the likelihood of one of the more common tumor types, including thymic epithelial neoplasms, lymphoproliferative tumors, germ cell tumor, or an endocrine lesion (thyroid or parathyroid). When a neuroblastic tumor is found in this region, its derivation from a germ cell tumor or spread from another site, including the sinonasal tract, should be prime considerations. Furthermore, considering the cellular nature of neuroblastomas, it becomes essential to take into account the possibility of other small round cell neoplasms like rhabdomyosarcoma and Ewing sarcoma. Regarding the latter, the nuclear positivity of thymic neuroblastoma for NKX2.2 can represent a diagnostic pitfall since it is a commonly used marker for Ewing sarcoma. This underscores the need for additional testing, including CD99 immunohistochemistry and FISH analysis for *EWSR1* rearrangement. In previously reported cases and our own experience, the definitive diagnosis of thymic neuroblastoma may only be achieved following complete surgical removal of the tumor, where a large panel of immunohistochemical stains and/or molecular analysis can be employed.

When exploring potential differential diagnoses for this tumor, we considered and eliminated other tumors with similar morphological characteristics. These included olfactory neuroblastoma (excluded based on clinical absence of nasal involvement) and Ewing sarcoma (due to lack of membranous CD99 staining and a negative *EWSR1* FISH test). While the overall characteristics align most closely with a neuroblastoma diagnosis, there are several features that do not fit the profile of pediatric-type neuroblastomas. Although our patient did not show evidence of the SIADH, prior cases of anterior mediastinal/thymic neuroblastomas in adult patients were associated with the syndrome. From a clinical perspective, paraneoplastic SIADH is more commonly associated with small cell carcinoma, especially of pulmonary origin, whereas the paraneoplastic manifestations of pediatric-type neuroblastoma include opsoclonus-myoclonus-ataxia syndrome and Kerner-Morrison syndrome [36, 37]. Additionally, the SIADH is frequently observed in cases of olfactory neuroblastoma [38]. The immunohistochemical staining for TdT is also atypical in neuroblastoma, although it is worth noting that TdT positivity has been rarely documented in neuroblastoma cases [23, 39]. Moreover, PHOX2B has emerged as a highly reliable immunohistochemical marker for pediatric-type neuroblastoma due to its high sensitivity and specificity when compared to other small round blue cell tumors commonly encountered in childhood. However, as many as 50% of adult neuroblastomas, including the current tumor, do not express PHOX2B, implying a potential divergence in the cell lineage from which these tumors originate [40].

In summary, we present a case of primary thymic neuroblastoma. Our report emphasizes the importance of a comprehensive diagnostic evaluation. Those patients with tumors confined to the anterior mediastinum, lacking involvement of adjacent organs, showing no evidence of metastasis, and are suitable candidates for complete surgical removal, experience more favorable outcomes and appear biologic distinct from pediatric-type neuroblastomas. Therefore, early diagnosis of these tumors is imperative. It is prudent to consider thymic neuroblastoma in cases where mediastinal tumors show neural differentiation, particularly in adult patients, the possibility of a germ cell tumor should be excluded because of differing treatments and prognoses.

## Data Availability

Data sharing is not applicable to this article as no datasets were generated or analysed during the current study.
